# Dataset on characterisation and stability of gingival retraction cord lignocaine–adrenaline nanogel optimised using central composite design

**DOI:** 10.1016/j.dib.2024.111028

**Published:** 2024-10-12

**Authors:** Min Mardhiyyah Azman, Muhammad Salahuddin Haris, Widya Lestari, Juzaily Husain, Omar Abdul Jabbar Abdul Qader, Wan Nor Hayati Wan Abd. Manan

**Affiliations:** a Department of Pharmaceutical Technology, Kulliyyah of Pharmacy, International Islamic University Malaysia, 25200 Kuantan, Pahang, Malaysia; b IKOP PHARMA Sdn. Bhd., 25200 Kuantan, Pahang, Malaysia; c Department of Fundamental Dental and Medical Sciences, Kulliyyah of Dentistry, International Islamic University Malaysia, 25200 Kuantan, Pahang, Malaysia; d Department of Restorative Dentistry, Kulliyyah of Dentistry, International Islamic University Malaysia, 25200 Kuantan, Pahang, Malaysia; e Department of Oral Diagnosis, College of Dentistry, Al-Mashreq University, Baghdad Airport Street; f Department of Prosthodontics, Kulliyyah of Dentistry, International Islamic University Malaysia, 25200 Kuantan, Pahang, Malaysia

**Keywords:** Lignocaine, Adrenaline, Nanotechnology, Central composite design, Haemostasis, Characterisation

## Abstract

This study aims to characterise and assess the stability of an optimised lignocaine–adrenaline nanogel using central composite design (CCD). Compatibility studies were conducted using Attenuated Total Reflectance-Fourier Transform Infrared (ATR-FTIR) and Ultraviolet–visible (UV–vis) spectroscopy. Eighteen lignocaine–adrenaline Nanoemulsion (LANE) formulations derived using CCD were characterised for particle size, polydispersity index (PDI), zeta potential and pH. All LANE formulations were transformed into lignocaine-adrenaline Nanoemulsion-based Gel (NBG) by adding 0.1 % Carbopol 940. Stability studies for LANE and NBG were conducted for 12 months storage at 25 °C. The results of long-term stability assessment of LANEs and NBGs were integrated with CCD predictions to produce the optimised NBG, lignocaine–adrenaline Nanogel. The optimised NBG model was validated in triplicates. The optimised NBG was subjected to 5000 rpm centrifugation for 30 min, repeated heating-cooling cycles (40 °C and 4 °C), and a freeze-thaw cycle (-5 °C and 25 °C). ATR-FTIR and UV–vis results indicated compatibility between lignocaine, adrenaline and the excipients. The viscosity of the nanogel corresponded to that of ferric sulphate solution (24 ± 1 mPa·s at 20 °C). The LANE and NBG formulations showed no drug precipitation or phase separation after the stability study. The optimised NBG had particle size (61.76 ± 0.25 nm), PDI (0.36 ± 0.01), zeta potential (−26.47 ± 0.02 mV) and pH (6.28 ± 0.02). The optimised NBG remained stable in stress-induced environments. CCD enabled optimisation of a stable NBG formulation.

## Graphical abstract

**Figure:**
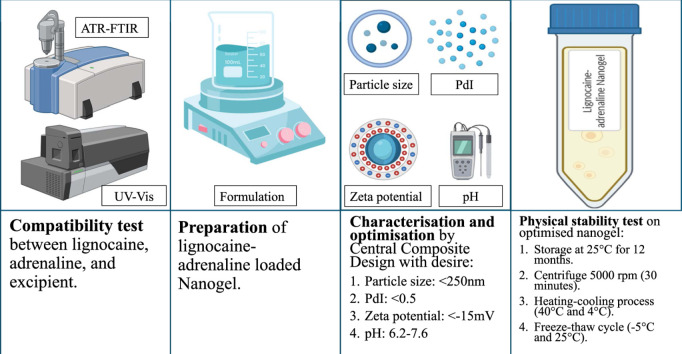

Specifications Table

**Table:** 

Subject	Materials science
Specific subject area	*Characterisation and stability testing on lignocaine-adrenaline nanoemulsion*
Type of data	Table, Image, Graph, Figure, Raw data
Data collection	Compatibility between lignocaine, adrenaline and excipients was assessed using Attenuated Total Reflectance-Fourier Transform Infrared (ATR-FTIR) spectroscopy (Perkin Elmer Spectrum, Connecticut, United States) and Ultraviolet–visible (UV–vis) spectrophotometer was used to confirm the compounds in mixture. The chosen nanoemulsion systems for drug solubilisation, including the optimised lignocaine-adrenaline nanoemulsion were formulated according to Central Composite Design (CCD). The formulations were prepared via spontaneous emulsification methods and the parameters were analysed using Malvern Zetasizer and Nano ZS Software (Nano-ZS, Malvern Instruments, Worcestershire, UK). The optimised formulation was generated using CCD, and stability testing was performed using a high-speed centrifuge (Supra 22 K, Hanil, South Korea), with repeated heating-cooling and freeze-thaw cycles*.*
Data source location	International Islamic University Malaysia (IIUM), Kulliyyah of Pharmacy, Kuantan Campus, Pahang, Malaysia.
Data accessibility	Repository name: Mendeley Data Data identification number: doi: 10.17632/4pjv66mhdw.1 Direct URL to data: data.mendeley.com/datasets/4pjv66mhdw/1
Related research article	*None*

## 1 Value of the Data

• The compatibility and detection of lignocaine and adrenaline in the mixture suggest that the formulation effectively encapsulated the active pharmaceutical ingredients (APIs) within the nanocarrier, thereby enabling the drugs to perform their intended functions within the oral tissues.
• Central composite design is an established experimental tool for designing and optimising formulations based on predefined criteria for dependent variables.
• The current investigation looks at the development and characterisation of selected LANE formulations and lignocaine–adrenaline-loaded NBGs, producing nanoparticles that are able to carry the active ingredient effectively and with good stability.
• Lignocaine–adrenaline nanogels demonstrated stability under high-force centrifugation and repeated heating-cooling and freeze-thaw cycles.

## 2 Background

Effective dental restorations require precise gingival management. Gingival retraction cords, often infused with haemostatic agents such as ferric sulphate, displace gingival tissue for improved visibility but can cause tissue irritation, bleeding, and discoloration [[1], [2], [3]]. The combination of lignocaine and adrenaline in the retraction cord enhances haemostasis and analgesia [4]. This research aimed to develop a dataset for stable lignocaine-adrenaline nanogel formulation to enhance efficacy, reduce tissue damage, and optimise haemostatic-analgesic effects during dental procedures.

## 3 Data Description

**Fig. 1:**
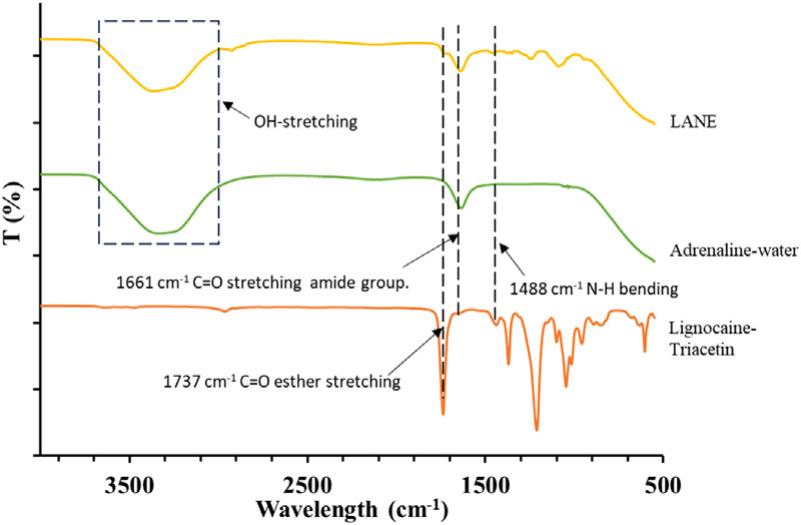
ATR-FTIR analysis of lignocaine–triacetin, adrenaline–water, and LANE.

**Fig. 2:**
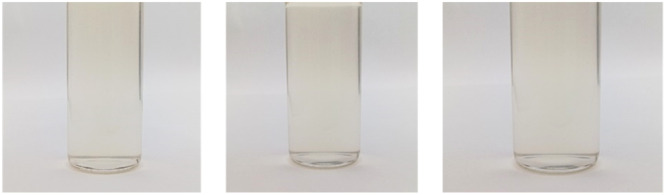
The optimised formulation showing no turbidity and good stability after centrifugation at 5000 rpm for 30 min, three consecutive heating-cooling cycles (40 °C and 4 °C) for 24 h, and a freeze-thaw cycle for 24 h (−5 °C and 25 °C). Formulation was tested in triplicates.

In the lignocaine–triacetin mixture spectra (Fig. 1), a notable ester stretching vibration was observed at 1737 cm^−1^, while all the three mixtures, including adrenaline-water and lignocaine–adrenaline nanoemulsion (LANE), exhibited notable amide group adsorption at 166 1 cm^−1^. The OH stretching region displayed overlapping features due to the presence of water, particularly with the adrenaline-water and LANE mixtures, complicating spectral interpretation in this region [5]. To corroborate the ATR-FTIR findings, UV–vis spectroscopy was used to analyse formulation samples. The absorbance values confirmed the presence of both lignocaine and adrenaline, which aligned with the concentrations predicted by the standard curves. CCD effectively predicted results that complied with the desired characteristics for the dependent variables (Y_1_-Y_4_) in the preparation of LANE suspensions. Drug inclusion was selected based on the phase diagrams' identification of oil/water (o/w) microemulsion (ME) areas shown in the research by Daryab et al. [6], where the systems consisted of oil/surfactant/oil with an extended o/w ME area and containing no more than 25% (w/w) surfactant and at least 5 % (w/w) oil combination [6]. The actual test data closely matched the predicted results, demonstrating the reliability of CCD in optimising formulation variables. The stability data of LANEs and NBGs after 12 months were quantified into the CCD system to produce an optimised nanogel formulation. The optimised nanogel exhibited stability in stress-induced environments, with no physical separation or segregation observed (Fig. 2). The CCD dataset has been deposited in Mendeley Data and is available at data.mendeley.com/datasets/4pjv66mhdw/1 [7]**.**

## 4 Experimental Design, Materials and Methods

### 4.1 Materials

The active substances lignocaine standard (Lidocaine) (Purity: ≥99 %), Adrenaline (L-Epinephrine) (Purity: ≥99 %) and Triacetin were supplied by Sigma-Aldrich (Darmstadt, Germany). Polysorbate 80, Polyethylene Glycol (PEG 400) and Carboxypoly-methylene (Carbopol 940) were all obtained from EvaChem (Selangor, Malaysia). Distilled water was prepared using Favorit Water Distiller by PLT Scientific Sdn. Bhd. (Selangor, Malaysia).

### 4.2 ATR-FTIR spectra of active substances mixture and lane compatibility

ATR-FTIR was used to assess the compatibility of the active pharmaceutical ingredients with their respective solvents. ATR-FTIR analysis was performed on mixtures of lignocaine–triacetin, adrenaline-water, and LANE. Spectra were acquired using an FTIR Spectrum Frontier (PerkinElmer Spectrum, Connecticut, United States) across the spectral range of 4000–550 cm^−1^, with a resolution of 4 cm^−1^.

### 4.3 UV–vis spectrophotometry

**Fig. 3:**
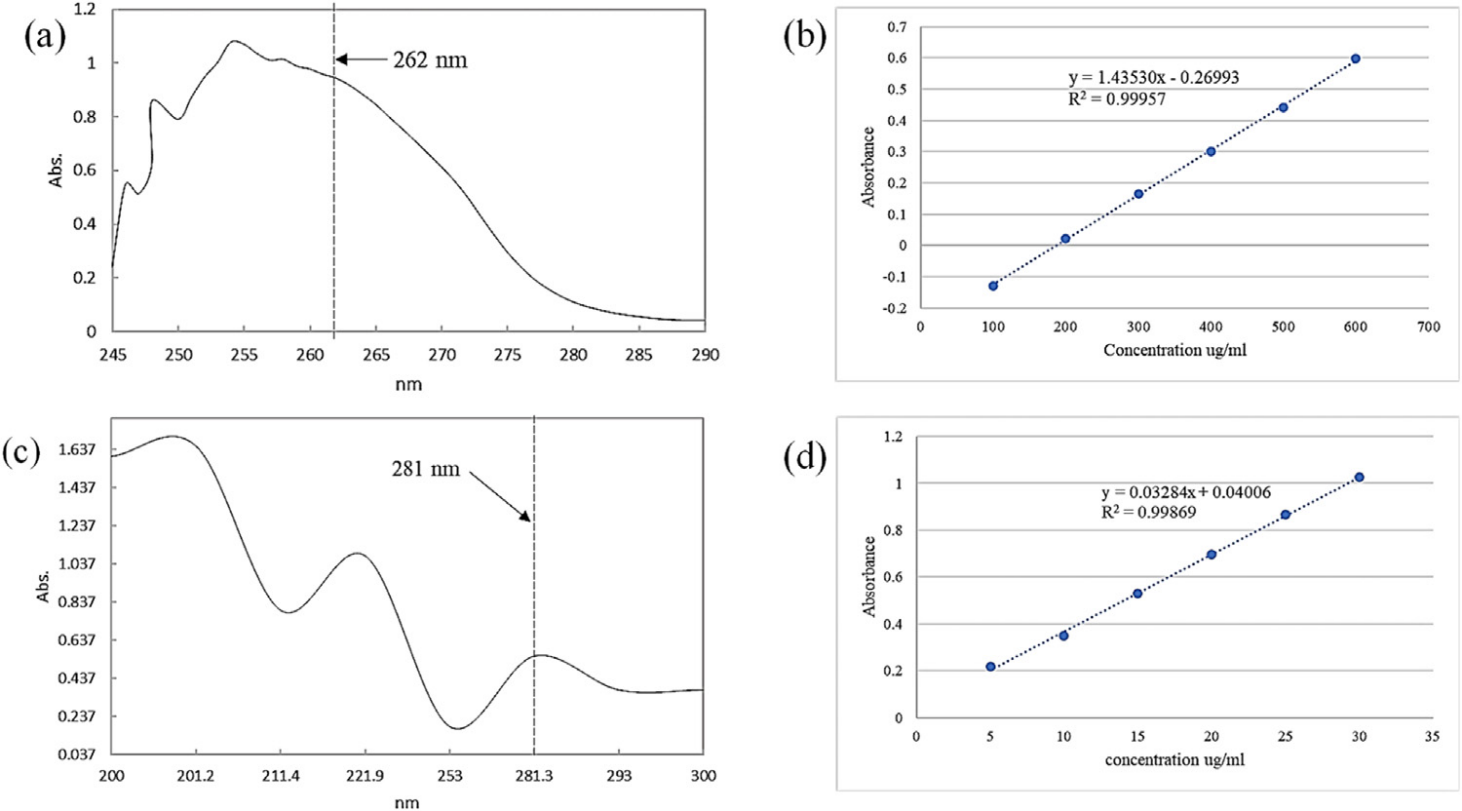
Representative UV spectra of two drugs for experimental formulation (a) Lignocaine in triacetin at λ_max_ 262 nm. (b) Calibration curve of lignocaine. (c) Adrenaline in distilled water at λ_max_ 281 nm. (d) Calibration curve of adrenaline.

Calibration curves function as a set of standard samples with known concentrations [8]. Six different concentrations of the lignocaine standard (100, 200, 300, 400, 500, and 600 µg/ml) and the adrenaline standard (5, 10, 15, 20, 25, and 30 µg/ml) were prepared using stock solutions to produce the calibration curve shown in Fig. 3(b and d). The absorbance of each concentration at its λ_max_ was determined using a fixed wavelength measurement technique. The calibration curve was plotted for absorbance against concentration. UV–vis spectroscopy was used in addition to ATR-FTIR analysis to confirm the presence of lignocaine and adrenaline in the LANE mixture. Lignocaine wavelength was set to 262 nm ± 1 [6,8] (λ max = 262 nm, Fig. 3(a)), and adrenaline wavelength was set to 282 nm ± 1 [9] (λ max = 281 nm, Fig. 3(c)). The regression line correlation coefficients (R^2^) for both lignocaine and adrenaline were 0.99957, (y-intercept= 0.26993) and 0.99869 (y-intercept= 0.04006) respectively (Fig. 3(b) and (d)).

### 4.4 The accuracy test

Accuracy was determined by calculating the recovery of predetermined amounts of LANE added into distilled water as dilution medium [10]. LANE suspension was diluted to a lignocaine concentration of 550 µg/ml and adrenaline concentration of 10 µg/ml, respectively. Analysis was performed in triplicate using UV–vis spectrophotometer. These concentrations were made with 110 % and 100 % equivalence to the respective absorbance readings of the standard curve for lignocaine at 500 µg/ml and adrenaline at 10 µg/ml. The recovery of the diluted LANE concentrations was 101.89 % for lignocaine and 99.59 % for adrenaline, respectively, with relative standard deviations (%RSD) of 0.69 and 0.46, respectively. Tests were conducted in compliance with the United States Pharmacopeia (USP)−857 guideline.

### 4.5 Constitution of the Chosen Nanoemulsion (NE) Systems for Drug Solubilisation and the Optimised Nanogel according to CCD

The experiments were designed with 3 factors and 4 central points, resulting in 18 runs. Each experiment was conducted independently based on the runs generated from CCD output. The optimised nanogel formulation was predicted by analysing Y_i_, comparing against actual test results of the selected formulation. CCD outputs were based on the variables in Table 1. Actual data from the runs were compared against CCD predictions to optimise nanogel formulation. Spontaneous emulsification methods were used to formulate the lignocaine–adrenaline loaded Nes; the concentrations of lignocaine and adrenaline were kept constant at 2 % and 0.001 % (1:100,000), respectively [11].

**Table 1:** Independent (Xi) and dependent (Yi) variables used in CCD with levels and optimisation criteria.

Independent variables (Xi)	Levels		
	Low	Medium	High
X_1_ (Triacetin) % (w/w)	4	5	6
X_2_ (Polysorbate 80) % (w/w)	12	14	16
X_3_ (PEG 400) % (w/w)	6	8	10
Dependent Variable (response) (Yi)	Optimisation Criteria		

Y_1_ (Z-Average (nm))	In Range (10.45 – 100.00 nm)		
Y_2_ (PDI)	Target (0.3)		
Y_3_ (Zeta Potential (mV))	Minimise (−26.5 mV)		
Y_4_ (pH)	In Range (6.10 – 6.46)		

### 4.6 Physical stability tests

**Fig. 4:**
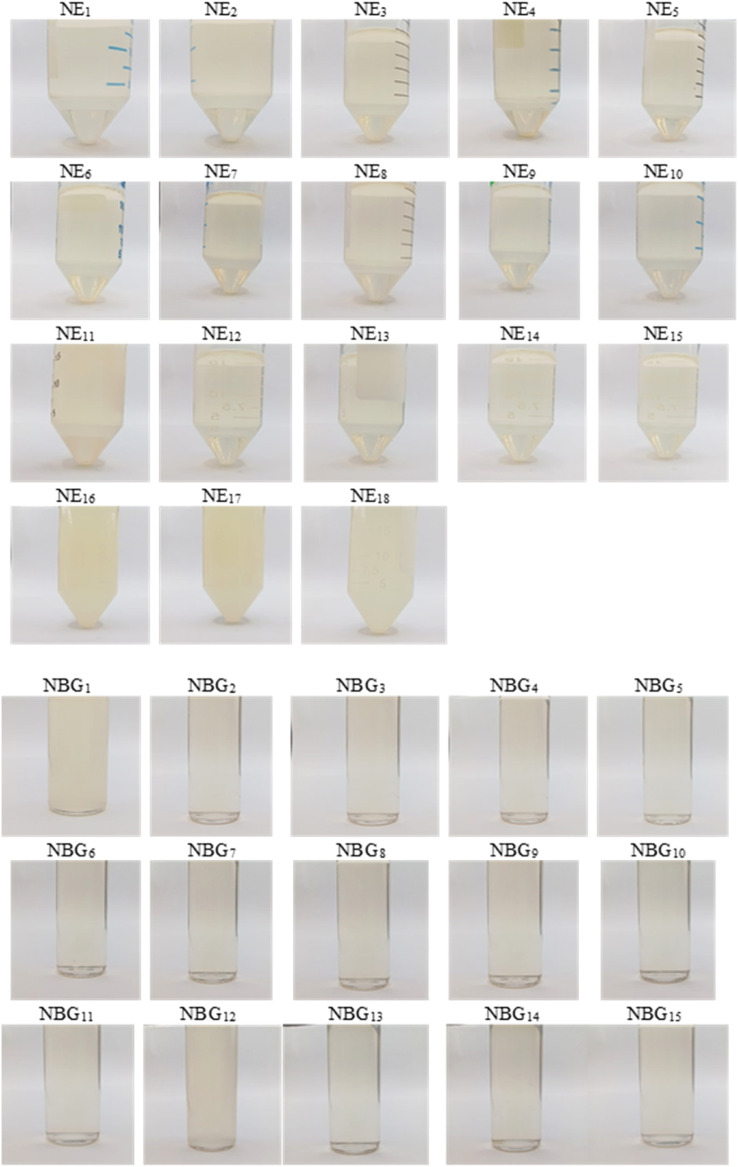
Stability of nanoemulsions (NEs) after 72 h of storage at room temperature. Besides NE_16_, NE_17_, and NE_18,_ all formulations showed no sedimentation or turbidity and were transparent. Following 12 months of storage at room temperature, no breakdown or textural shift was noted in any of the nano-based gel (NBG) formulations under investigation, apart from NBG_1_ and NBG_12_.

The fluid NEs were kept in a 50 ml sealed conical centrifuge tube at 25 °C for 72 h. During this time, any physical changes such as turbidity, phase separation, drug precipitation, and colour changes were monitored [6]. The stable fluid NEs were transformed into nano-based gels (NBGs) and left for 12 months to assess for long-term physical stability (Fig. 4). NBGs that did not undergo physical changes were analysed, and the data was included in the CCD analysis to produce the optimised NBG. The optimised NBG was subjected to 30 min of centrifugation at 5000 rpm using high-speed centrifuge (Supra 22 K, Hanil, South Korea), repeated heating and cooling cycles for 24 h at 40 °C and 4 °C, as well as 24 h of freeze-thaw (FT) cycle at −5 and 25 °C [6,12,13].

## Limitations

The research design space was limited to testing four excipients: Triacetin (4–6 %), Tween 80 (12–16 %), PEG 400 (6–10 %), and Carbopol 940 (0.1 %) to achieve a viscosity of 24 mPa·s, which was necessary to match the performance of ferric sulphate. Variables outside this design space were not tested due to concerns with instability [6].

## Ethics Statement

This research work does not involve human subjects, animal experiments, or any data collected from social media platforms.

## CRediT authorship contribution statement

**Min Mardhiyyah Azman:** Conceptualization, Methodology, Investigation, Formal analysis, Software, Data curation, Validation, Visualization, Writing – original draft. **Muhammad Salahuddin Haris:** Conceptualization, Methodology, Investigation, Validation, Visualization, Writing – review & editing. **Widya Lestari:** Investigation, Validation, Visualization. **Juzaily Husain:** Investigation, Validation, Visualization. **Omar Abdul Jabbar Abdul Qader:** Investigation, Validation, Visualization. **Wan Nor Hayati Wan Abd. Manan:** Conceptualization, Methodology, Investigation, Validation, Visualization, Supervision, Project administration, Writing – review & editing.

## Data Availability

Mendeley DataCharacterisation and stability testing on lignocaine-adrenaline nanosuspension (Original data)
